# Surgery combined with adjuvant radiation and chemotherapy prolonged overall survival in stage IVC anaplastic thyroid cancer: a SEER-based analysis

**DOI:** 10.1007/s12020-023-03662-7

**Published:** 2024-01-06

**Authors:** Ying Yin, Linhe Wang, Chiming Huang

**Affiliations:** Department of Thyroid and Hernia Surgery, Guangdong Provincial People’s Hospital (Guangdong Academy of Medical Sciences), Southern Medical University, Guangzhou, 510080 China

**Keywords:** Anaplastic thyroid carcinoma, Surgery, Radiation therapy, Chemotherapy

## Abstract

**Background:**

Anaplastic thyroid carcinoma (ATC) is a rare but aggressive malignancy, which accounts for only 1–2% of all thyroid cancers. The median overall survival (OS) time for all stages patients is at about 5 months. The benefit of surgery combined with adjuvant radiation and chemotherapy in stage IVC anaplastic thyroid cancer is still controversial. The aim of this study is to investigating surgery combined with adjuvant radiation and chemotherapy and survival outcomes in stage IVC ATC patients.

**Method:**

Anaplastic thyroid carcinoma patients from the Surveillance, Epidemiology, and End Results database from 2004 to 2016 were used to conduct a cross-sectional study in the analysis. The endpoint of this study was overall survival.

**Results:**

The median OS of the overall population was 2.0 months. Multivariate analysis showed that age (<67 vs. ≥67 years old, *P* = 0.017, HR = 1.355, 95% CI: 1.057–1.738), tumor size (<7 cm vs. ≥7 cm, *P* = 0.001, HR = 1.579, 95% CI: 1.202–2.073), Surgery (thyroidectomy vs. non-surgery, *P* < 0.001, HR = 0.554, 95% CI: 0.401–0.766), radiation therapy (*P* < 0.001, HR = 0.571, 95% CI: 0.445–0.733) and chemotherapy (*P* = 0.003, HR = 0.684, 95% CI: 0.531–0.881) were independent prognostic factor for worse OS in stage IVC ATC patients. Surgery combined with adjuvant radiation and chemotherapy exhibited the better overall survival time for 4 months.

**Conclusions:**

Surgery combined with adjuvant radiation and chemotherapy can improve overall survival in stage IVC ATC patients. We recommend surgical approach with fully evaluation combined with radiation therapy and chemotherapy for selected stage IVC ATC patients.

## Introduction

Anaplastic thyroid carcinoma (ATC) is a rare but aggressive malignancy, which accounts for only 1–2% of all thyroid cancers [1]. The median overall survival (OS) time for all stages patients are at about 5 months and a 1-year overall survival of 20% [2]. Furthermore, ATC is usually diagnosed with clinical symptoms including dyspnea, dysphagia, rapidly size-increasing neck mass and so on, while half of ATC patients would have distant metastasis at initial diagnosis [3]. In 2021, American Thyroid Association updated the Guidelines for Management of Patients with Anaplastic Thyroid Cancer. Surgical option was still considered as the primary treatment approach for early-stage ATC (stages IVA and IVB) patients. Combined with adjuvant radiation and chemotherapy, long time survival may be possible [4].

However, for tumors at stage IVC, surgery was not the first option for the patients, while radiotherapy (RT), chemotherapy (CT) and target therapy may be the favorable treatment for stage IVC ATC patients. However, in some recent retrospective studies, the results demonstrated that stage IVC ATC patients may be beneficial for the early surgery, followed by the RT, CT and target therapy [5, 6]. The role of surgery combined with adjuvant radiation and chemotherapy in stage IVC anaplastic thyroid cancer has not been well defined. We hypothesized that stage IV ATC patients may gain the benefit of surgery combined with adjuvant radiation and chemotherapy. To verify this hypothesis, we conducted this study by using the Surveillance Epidemiology and End Results (SEER) database to explore the correlation between surgery combined with adjuvant radiation and chemotherapy and survival outcomes in stage IVC ATC patients and to determine whether surgery should be recommended for stage IVC ATC patients.

## Methods

### Data source and patients

**Fig. 1:**
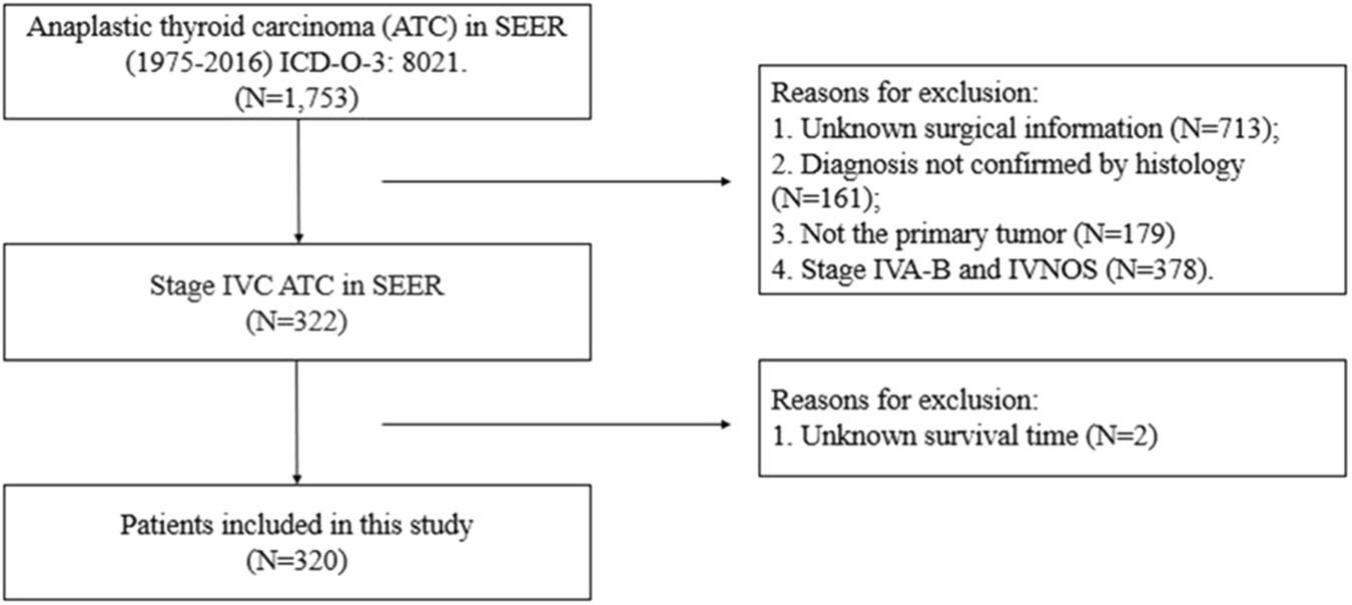
Flowchart summarizing patient enrollment

We used the SEER database, the population-based registry for cancers in the United States, which collects cancer incidence data from population-based cancer registries covering approximately 34.6 percent of the U.S. population (https://seer.cancer.gov/). Patient information was obtained from their medical records. This study was exempted by the ethics committee of the Guangdong Provincial People’s Hospital because our data were from the SEER database, which is open to the public. All data and parameters were utilized from SEER Program Research Data (1973–2015), NCI, Division of Cancer Control and Population Sciences, Surveillance Research Program. We obtained data from the SEER*Stat software, version 8.3.6. Morphology code 8021, used to identify ATC, in the International Statistical Classification of Diseases for Oncology, 3rd Edition (ICD-O-3), was selected in the study. Patients were included according to the following criteria: (I) histopathological confirmed diagnosis of ATC between 2004 and 2016; and (II) primary diagnosis of ATC. Some patients were excluded from our study basing on any one of the following criteria: (I) patients with unknown follow-up data and detailed surgical information in the database; (II) patients with stage IVA-B and not specified disease (stage IVNOS) according to the AJCC 6th staging manual; (III) mixed histologic types of thyroid malignancy, shown in Fig. 1.

### Data analysis

The characteristics of patients (age, sex, race, married status and insurance status), tumor characteristics (tumor size and metastasis details), treatment (surgery, radiotherapy and chemotherapy), cause of death, survival duration and vital status were gathered from the database. Besides, surgery was defined as (I) thyroidectomy; (II) less than thyroidectomy (with residual thyroid tissue), (III) non-surgery and (IV) unknown. The primary outcome was OS. Age and tumor size were divided into two groups based on the median number.

### Statistical analysis

Statistical analysis was performed by using IBM SPSS statistics 26.0 software (SPSS, Armonk, New York, USA). *P* values < 0.05 were considered statistically significant. Survival analyses were carried out with respect to cancer-specific survival (CSS) and overall survival (OS). The OS and CSS were calculated by the Kaplan–Meier method and the significance of differences between two or more survival curves were assessed by using the log-rank test. Cox proportional hazards model was used for univariable and multivariable analyses to determine the risk factors. HR and 95% confidence interval (CI) were calculated.

## Results

### Demographic and clinicopathological characteristics

The clinicopathological characteristics of the 320 study patients are listed in Table 1. The median age at diagnosis was 67 years old. A total of 241 patients were white (75.3%), and the female to male ratio is 1.32:1. More than half patients were married and insured, 57.8% and 61.9%, respectively. The median tumor size was 7.0 cm. 62 (19.3%) stage IVC ATC patients underwent total thyroidectomy, while 203 patients did not receive surgery or the status was unknown. More than half of the patients received RT (51.5%), and 39.7% of the patients received CT.

**Table 1:** Univariate and multivariate analyses of OS

Factor	Number	Overall Survival (OS)					
		Univariate			Multivariate		
		*P* value	HR	95% CI	*P* value	HR	95% CI
Age		0.028	1.338	1.031–1.736	0.017	1.355	1.057–1.738
<67	151						
≥67	169						
Race		0.558	0.92	0.697–1.215			
white	241						
nonwhite	79						
Sex		0.752	0.959	0.743–1.240			
female	182						
male	138						
Tumor size(cm)							
Reference: <7	110	0.001			0.001		
≥7	130	0.003	1.533	1.167–2.066	0.001	1.579	1.202–2.073
Unknown	80	0.001	1.76	1.255–2.467	0.001	1.685	1.225–2.318
Surgery at the primary site							
Reference: None	197	0.002			0.003		
Less than thyroidectomy	55	0.217	0.815	0.589–1.128	0.281	0.841	0.615–1.152
Thyroidectomy	62	<0.001	0.526	0.377–0.735	<0.001	0.554	0.401–0.766
Unknown	6	0.194	0.536	0.210–1.372	0.209	0.562	0.229–1.380
Positive lymph nodes							
Reference: none or negative	80	0.892					
Positive	132	0.81	0.966	0.729–1.280			
Unknown	46	0.815	1.049	0.701–1.570			
Radiation recode		<0.001	0.579	0.448–0.748	<0.001	0.571	0.445–0.733
no/unknown	156						
yes	164						
Chemotherapy recode		0.002	0.657	0.505–0.854	0.003	0.684	0.531–0.881
no/unknown	193						
yes	127						
Marital status		0.398	0.894	0.688–1.160			
Married	185						
Unmarried and others	135						
Insurance status		0.934	1.011	0.780–1.3101			
Insured	198						
Uninsured and others	122						

### Prognostic factors for OS

The median OS of the overall population was 2.0 months. The 1-year OS rate was 9.5%. A total of 311 (97.2%) stage IVC ATC patients died during the follow-up period. Among them, 295 (92.2%) patients died because of ATC, and the other 16 (5.0%) stage IVC ATC patients died of other causes.

In the univariate analyses, as shown in Table 1, we found that prognostic factors for OS in stage IVC ATC patients were significantly related to age (*P* = 0.028), tumor size (*P* = 0.001), surgery (*P* = 0.002), radiation therapy (*P* < 0.001), chemotherapy (*P* = 0.002). There were no significant differences in other clinicopathological factors such as race, sex, married status, tumor extension and lymph node metastasis. For the multivariate analysis, after adjusting for other available variables, it revealed age (<67 *vs*. ≥67 years old, *P* = 0.017, HR = 1.355, 95% CI: 1.057–1.738), tumor size (<7 cm *vs*. ≥7 cm, *P* = 0.001, HR = 1.579, 95% CI: 1.202–2.073), Surgery (thyroidectomy *vs*. non-surgery *P* < 0.001, HR = 0.554, 95% CI: 0.401–0.766), radiation therapy (*P* < 0.001, HR = 0.571, 95% CI: 0.445–0.733) and chemotherapy (*P* = 0.003, HR = 0.684, 95% CI: 0.531–0.881) were independent prognostic factor for worse OS in stage IVC ATC patients, as shown in Table 1.

**Fig. 2:**
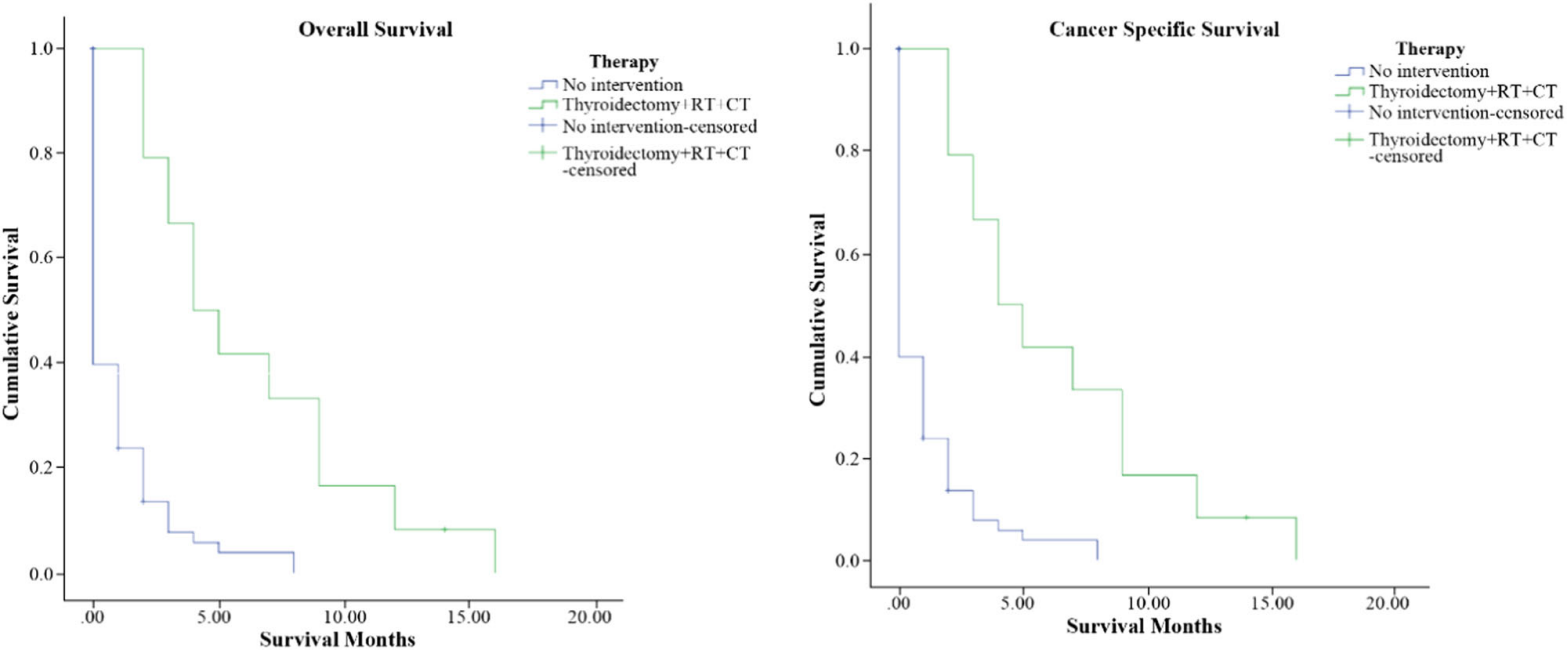
OS and CSS of Stage IVC ATC patients according to surgery versus nonsurgical treatment (*p* < 0.01) (RT radiation therapy, CT Chemotherapy)

**Fig. 3:**
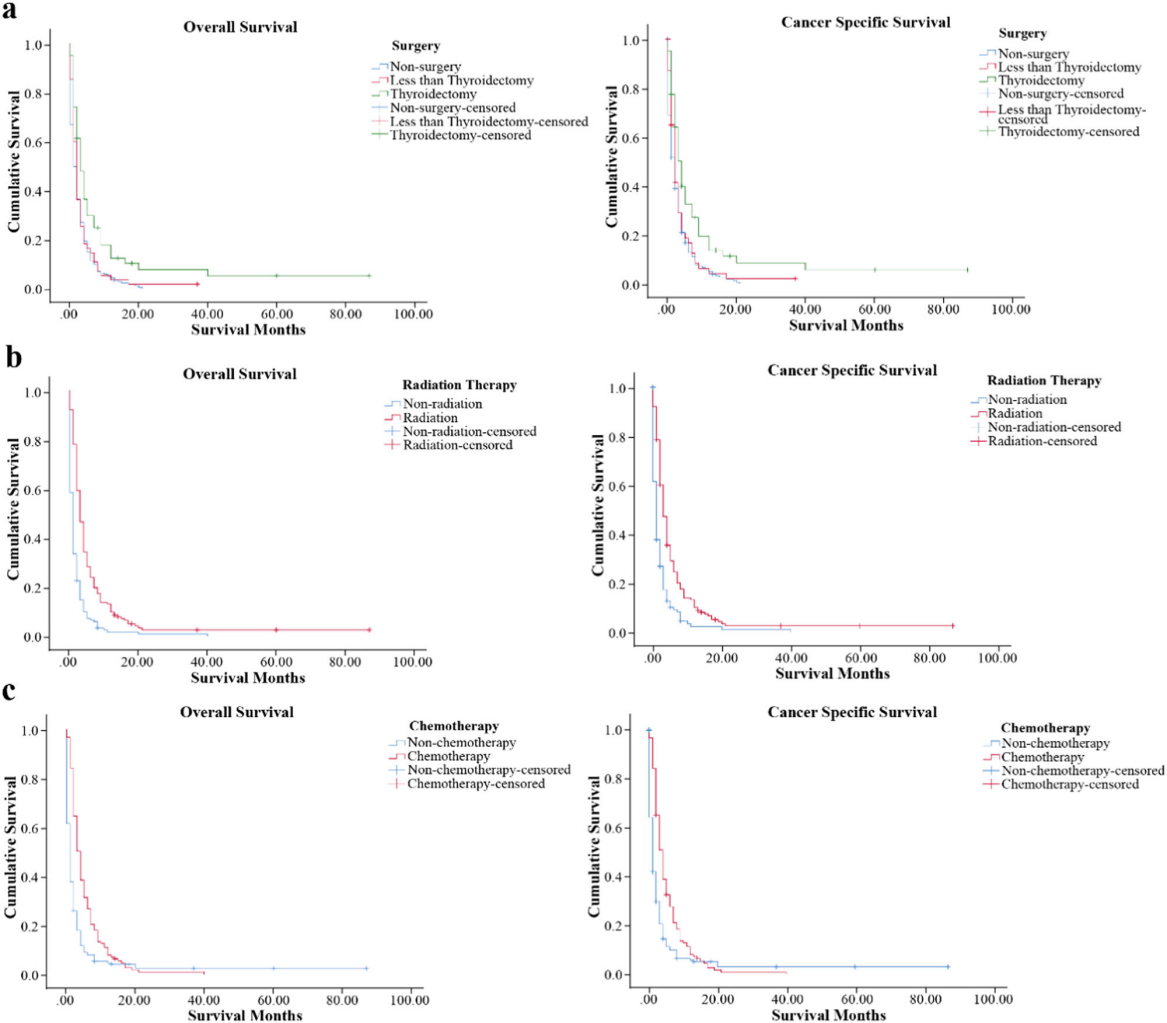
OS and CSS of Stage IVC ATC patients according to **a** surgery at the primary site, **b** the use of radiation therapy and **c** the use of chemotherapy (*p* < 0.01)

**Fig. 4:**
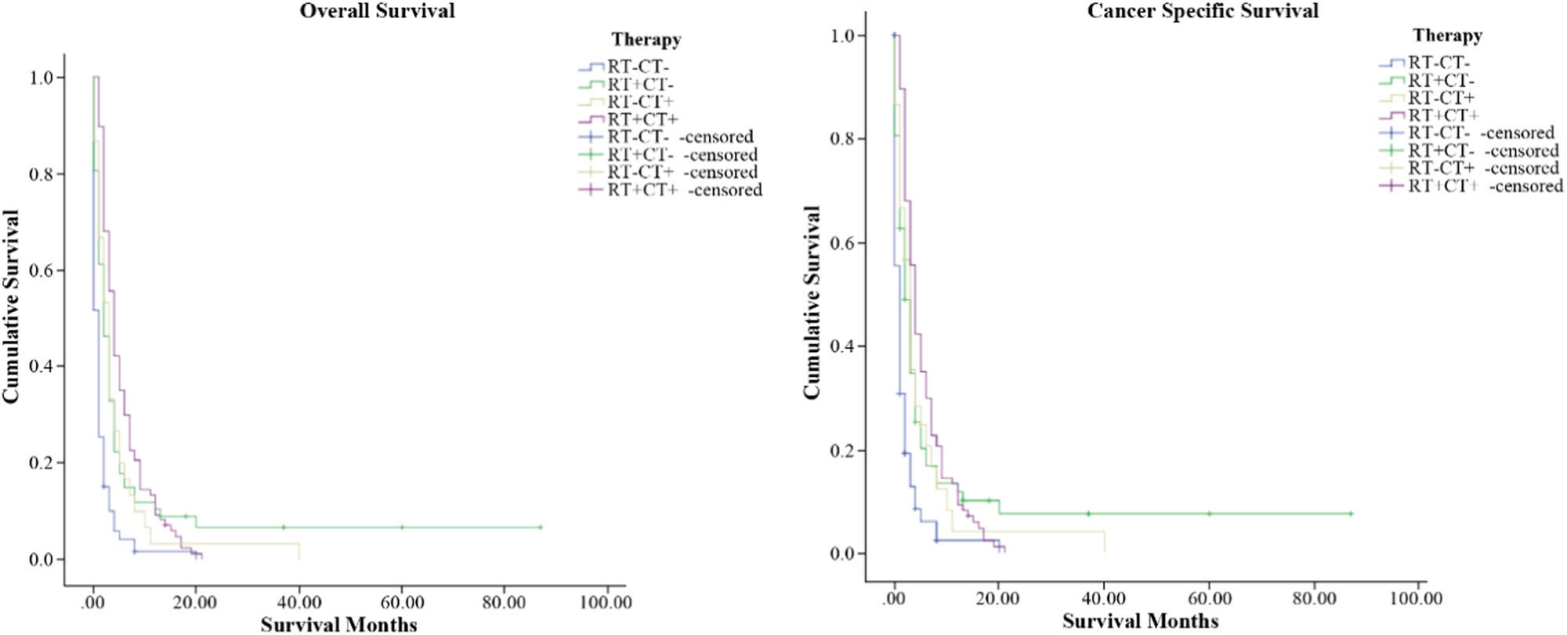
OS and CSS of Stage IVC ATC patients according to the different treatment approaches after thyroidectomy (*p* < 0.01) (RT radiation therapy, CT chemotherapy)

117 stage IVC ATC patients received thyroid surgery. Among them, 24 stage IVC ATC patients received thyroidectomy combined with RT and CT. The median survival time for patients who received thyroidectomy combined with RT and CT was 4 months, and this was significantly prolonged the survival time of patients who with no-cancer directed surgery (*P* < 0.001) (shown in Fig. 2). Patients who undergo thyroidectomy have significantly better OS and CSS than those who undergo less than thyroidectomy and No-surgery (*P* < 0.001) (shown in Fig. 3a). As for radiation therapy, survival was better for patients receiving radiation therapy than patients who did not receive radiation therapy (shown in Fig. 3b). Also, the chemotherapy was similarly beneficial to survival for stage IVC ATC patients (shown in Fig. 3c). For postoperative treatment options, the median survival time for patients who received radiation therapy alone after surgery and postoperative chemotherapy alone were also prolonged, both achieving 3 months (*P* < 0.001) (shown in Fig. 4).

## Discussion

According to 2021 American Thyroid Association Guidelines for Management of Patients with Anaplastic Thyroid Cancer, for surgical decision making, the benefit and the morbidity should be balanced, also the patient-anticipated prognosis should be considered, especially for presence of distant metastasis (stage IVC) ATC patients [4]. In the SEER database, the median overall survival time was 2.0 months, similarly with the historical median survival [7].

In our study, multivariate analysis shows age ≥67 years old and tumor size ≥7 cm were independent prognostic factor for worse OS in stage IVC ATC patients, similar with the identified prognostic factors in previous studies [8–10]. Furthermore, older than the median age is widely adopted as clinically important prognostic factor in ATC studies. The age between 60 and 68 years old was used as the threshold of the prognostic factor in the previous studies, also 67 years old was set as threshold in this study [11–13]. The prognosis of people older than the median age was worse than that of people younger than the median age in our study. It has been reported that small tumors indicated better prognosis [14], we found that tumor size was correlated with prognosis of stage IVC ATC patients (<7 cm *vs*. ≥7 cm, *P* = 0.001, HR = 1.579). It is assumed that large size may lead to more emergent situations and deadly complications during the treatment process.

We further determined the correlation between surgery and better survival in stage IVC ATC patients, but few studies were concerning the specific stage IVC ATC cohorts [11]. Our results showed better prognosis in patients undertaking complete thyroid resections compared with those patients who did not receive cancer-specific surgery. In previously studies, Huang et al. [15] reported that total thyroidectomy may not beneficial to patients with very early stage or distant metastasis, by using SEER database in 2019. In contrast, Song et al. [11] demonstrated in 2020 that treatment consisting of total thyroidectomy were beneficial for patients with metastatic anaplastic thyroid carcinoma, with the usage of the SEER database data from 1998 to 2015. Based on the SEER database, the benefit of the surgery for stage IVC ATC patients was still controversial. In the large cohort study of 677 patients in Japan [14], for 233 stage IVC ATC patients, radical surgery achieved better survival time compared with the palliative surgery or non-surgery. For further discussing the difference among surgical strategies, Brignardello et al. [6] reveal that for stage IV-B and IV-C ATC patients, complete thyroid resection, or even resection with minimal macroscopical residual tumor were associated with longer survival time than partial thyroidectomy (6.57 months [CI 5.52–12.09] vs. 3.25 months [CI 0.66–4.80]).

Besides, in our study, radiation therapy and chemotherapy in stage IVC ATC patients were correlated with better OS and CSS. It is consistent with the finding of the study based on data from the National Cancer Database (NCDB). To evaluate the efficiency of radiation therapy and chemotherapy in ATC patients, Tian et al. [16] found that postoperative combination of radiation therapy and chemotherapy was associated with better survival than the radiation therapy alone. The role surgery combined with adjuvant radiation and chemotherapy also emphasized. Kebebew et al. [9] and Swaak-Kragten et al. [17] both demonstrated that thyroidectomy combined with radiation therapy and chemotherapy alleviated the cause-specific mortality rate in stage IVC ATC patients. It should be noted that although the adverse effect of subsequent radiation therapy and chemotherapy should be considered, the benefit of surgery combined with adjuvant radiation and chemotherapy was significant.

Clinically, the usual postoperative adjuvant treatment for ATC patients includes local RT, with or without simultaneous or sequential CT. From the ESMO clinical practice guideline, we can know that the recommended radiation dose range for ATC patients is between 20 and 70 Gy [18]. For patients receiving palliative care, 20 Gy in 5 fractions to 30 Gy in 10 fractions is most common. The recommended radiation dose for curative treatment usually exceeds 45–50 Gy. Higher doses have a positive effect on local control, reduce the risk of local recurrence, thereby improving overall survival, and do not necessarily mean higher toxicity [19]. CT is often used in combination with adjuvant RT. Recommended CT regimens include paclitaxel against the cell division machinery or doxorubicin against the DNA repair pathway alone, or in combination, such as carboplatin/paclitaxel and docetaxel/doxorubicin [20]. The role of CT in ATC is still controversial, and its survival benefits and treatment toxicity are difficult to balance. For ATC patients with stage IVC, our study suggest that even CT alone can improve overall survival rate in the short term.

In 2021 American Thyroid Association Guidelines for Management of Patients with Anaplastic Thyroid Cancer, acquiring genomic information and performing target therapy were recommended [4]. BRAF V600E mutation should be rapidly assessed once ATC is diagnosed [21]. Recently, a phase II, open-label trial about dabrafenib (BRAF inhibitor) and trametinib (MEK inhibitor) combination therapy revealed that the overall response rate was 69% and 12-month overall survival rate was 80% [22]. Besides, another trial concerning Lenvatinib demonstrated that half of the patients experienced tumor shrinkage and median overall survival was 3.2 months (95% CI, 2.8 to 8.2) with the implement of the Lenvatinib, indicating that monotherapy may not suitable for ATC patients [23]. Adjuvant immunotherapy combined with target therapy was also explored and got significant results [24]. Therefore, novel combination therapies are needed to improve the outcomes of ATC patients. The prolonged survival time after thyroidectomy combined with radiation therapy and chemotherapy may provide the opportunity for patient to receive the target therapy and immunotherapy.

However, some questions still remain unclear due to limitations of the SEER database. Firstly, the results of the preoperative examination are not available to evaluate the fitness of the surgery strategy, including the ultrasound or the fine needle aspiration biopsy. Secondly, detailed information on the regimens, drugs, and sequences of radiotherapy and chemotherapy is not available in the SEER database. In this regard, some bias might influence the final conclusion in this study.

## Conclusion

In conclusion, we analyzed 320 stage IVC ATC patients in the SEER database, and our results demonstrated that surgery combined with adjuvant radiation and chemotherapy can improve overall survival in stage IVC ATC patients. We recommend surgical approach with fully evaluation combined with radiation therapy and chemotherapy for selected stage IVC ATC patients in the future. Multi-center, prospective trials are needed to fully evaluate the benefits of surgery combined with radiation therapy and chemotherapy for anaplastic thyroid carcinoma.
